# Multi-Omics and RNAi Reveal a Role for the Ubiquitin-Proteasome Pathway in Muscle Fiber Development of Common Carp (*Cyprinus carpio*)

**DOI:** 10.3390/ijms27219624

**Published:** 2026-10-27

**Authors:** Yating Gong, Mingkun Luo, Xianbo Zhang, Ning Qin, Lanmei Wang, Wenbin Zhu, Jianjun Fu, Zaijie Dong

**Affiliations:** 1 Wuxi Fisheries College, Nanjing Agricultural University, Wuxi 214081, China; 2 Key Laboratory of Freshwater Fisheries and Germplasm Resources Utilization, Ministry of Agriculture and Rural Affairs, Freshwater Fisheries Research Center, Chinese Academy of Fishery Sciences, Wuxi 214081, China; 3 Fisheries Research Institute, Guizhou Academy of Agriculture Sciences, Guiyang 550025, China

**Keywords:** *Cyprinus carpio*, muscle fiber development, ubiquitin proteasome, transcriptome, proteome, RNAi

## Abstract

To investigate the molecular mechanisms of muscle fiber development underlying locomotion differences between two common carp (*Cyprinus carpio*) strains cultured in a rice-fish symbiosis system, we conducted comparative transcriptomic and proteomic analyses of muscle tissues collected from indolent Congjiang (CJ) and active FFRC No. 2 (FR) strains cultured under identical paddy field conditions. RNA-seq revealed 3708 differentially expressed genes (DEGs), and proteomic analysis identified 108 differentially expressed proteins (DEPs). Functional enrichment and protein-protein interaction (PPI) network analyses highlighted key genes (*psma*3, *ppargc*1*a*, *ube*3*a*) and proteins (Myh, Ckmt2b, Suclg2) associated with proteasome activity, adipocytokine signaling, and ubiquitin-mediated protein degradation. Quantitative PCR (RT-qPCR) analysis of ubiquitin-proteasome pathway-related genes confirmed significantly higher expression levels in the muscle tissue of CJ compared with FR. After a 21-day interference experiment, *ube*3*a* knockdown increased average muscle fiber area and diameter and reduced muscle fiber density in juvenile CJ, indicating that *ube*3*a* knockdown might promote muscle development in common carp. Subsequent swimming performance measurements can be conducted to further explore the impact of *ube*3*a* on fish locomotion. These results provide molecular insights into fish locomotion capacity related to behavioral adaptation in agroecological systems.

## 1. Introduction

Rice-fish co-culture combines rice cultivation and fish farming in paddy fields, fully utilizing the ecological environment to achieve mutual benefits from the symbiosis between fish farming and rice cultivation [1]. The rich resources of aboriginal and improved common carp strains in China provide a wide range of strains that can be used in rice-fish symbiotic systems [2]. Because of their distinct characteristics, various common carp strains exhibit different behaviors and performance in rice fields. As integrated rice planting and fish farming models are promoted, the physiological and behavioral responses of fish in rice paddy ecosystems are receiving increasing attention. The Congjiang strain of common carp, adapted to the GIAHS site in Congjiang County, Qiandongnan Miao and Dong Autonomous Prefecture, Guizhou Province, China, is well suited to shallow paddy field waters because of its relatively low locomotor activity (locally known as “dull carp”), although its growth rate is low. In contrast, the FFRC No. 2 strain of common carp, a nationally authorized improved aquaculture variety (registration number GS-01-003-2017) with high growth and survival rates [3,4], shows more active locomotor behavior, leading to a higher likelihood of escape when cultured in paddy fields. Because locomotor ability affects fish retention in paddy fields, differences in locomotion among strains have gradually attracted the attention of both farmers and researchers.

In our previous research, we found that muscle characteristics affected the movement capacity of fish [5]. However, the regulatory mechanism of locomotion remains unclear. The ubiquitin-proteasome pathway (UPP) is a core regulatory cascade that governs muscle growth, fiber remodeling and functional differentiation in vertebrates. It precisely controls muscle cell proliferation, differentiation, and morphological characteristics by mediating targeted protein degradation. Although the UPP has been widely reported in mammalian muscle development, its functional role in fish muscle fiber formation and the regulation of locomotor traits is still poorly understood. Exploring UPP-related key genes can provide novel insights into the phenotypic divergence of fish muscle and movement. High-throughput multi-omics technology is an effective tool for screening trait-related core genes and pathways in aquatic animals [6,7,8]. Based on previous phenotypic findings, this study further applied integrated transcriptomic and proteomic analysis to screen candidate genes associated with carp muscle fiber development and detected the expression levels of selected genes to evaluate their effects.

To verify the biological function of target genes, RNA interference (RNAi), a mature and efficient gene silencing technology widely applied in functional research on fish, was used for in vivo functional verification [9]. Song et al. [9] successfully inhibited gonadal development in common carp by injecting *igf*3-dsRNA into the fish. In addition, Acosta et al. [10] reported that microinjection of tilapia myostatin-dsRNA into early zebrafish embryos led to gene silencing and caused the hatched zebrafish to exhibit obvious hypertrophy. These studies have demonstrated that it is feasible to use dsRNA interference technology to study gene function in fish. In this study, *ube*3*a*-dsRNA was synthesized in vitro, and short-term in vivo *ube*3*a* interference experiments were conducted. After three weeks of *ube*3*a*-dsRNA intervention, RT-qPCR was used to detect the expression of *ube*3*a* and its upstream and downstream genes. Western blotting (WB) and immunofluorescence (IF) were also used to detect Ube3a protein expression levels after interference. Muscle tissue was collected for histological examination by H&E staining, followed by analysis of muscle fiber characteristics to study the role of *ube*3*a* in muscle fiber development in common carp. The aim of this study was to identify key candidate genes and proteins related to locomotion capacity and to explore their potential regulatory mechanisms. The results of this study will lay the foundation for exploring the complex molecular regulatory network of the differences in locomotion between Congjiang strain common carp and FFRC No. 2 strain common carp, and will also provide basic data and reference for the genetic breeding of new aquaculture strains in the future.

## 2. Results

### 2.1. Identification of DEGs and Enrichment Analysis

After stringent quality evaluation and data filtering, a total of 241.78 million clean reads of the transcriptome (36.27 Gb high-quality sequences) were retained, ranging from 5.67 to 6.28 Gb per sample (Table S1). Among them, the mapping rate of clean reads to the reference genome (*Cyprinus carpio*, GCF_000951615.1) ranged from 81.10% to 84.01% (Table S1). Using StringTie (v1.3.5) software, all high-quality reads were aligned and assembled into 52,384 transcripts. These transcripts were further clustered based on nucleotide sequence identity, resulting in 44,222 unigenes. High correlations were observed among biological replicates, implying that the data were reliable.

**Figure 1:**
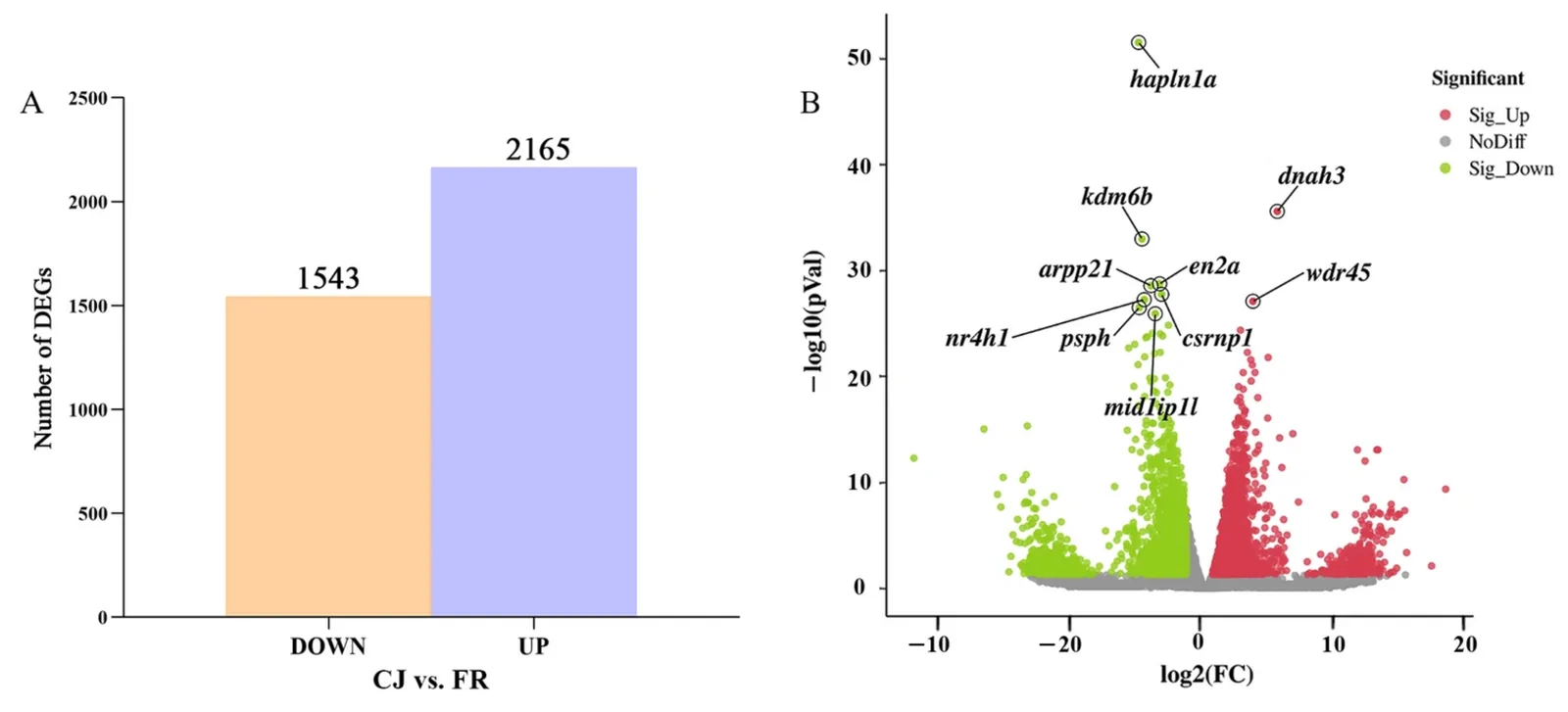
(**A**) Numbers of up- and down-regulated genes in the comparison of CJ versus FR. (**B**) Volcano map of differentially expressed genes. Red and green spots indicate significantly up- and down-regulated genes, respectively.

In total, 3708 differentially expressed genes (DEGs) were identified between CJ and FR, including 2165 up-regulated genes and 1543 down-regulated genes (Figure 1A). The volcano plot further illustrated the expression trend of DEGs (Figure 1B). It was found that genes such as *dnah*3 (Dynein axonemal heavy chain 3) and *wdr*45 (WD repeat domain 45) were highly expressed in CJ, while genes such as *hapln*1*a* (Hyaluronan and proteoglycan link protein 1a), *kdm*6*b* (Lysine demethylase 6b) and *mid1ip*1 (MID1 interacting protein 1) were highly expressed in FR. The *dnah*3 belongs to the motor protein family, and the gene family members encode large proteins associated with microtubule motor protein complexes.

**Figure 2:**
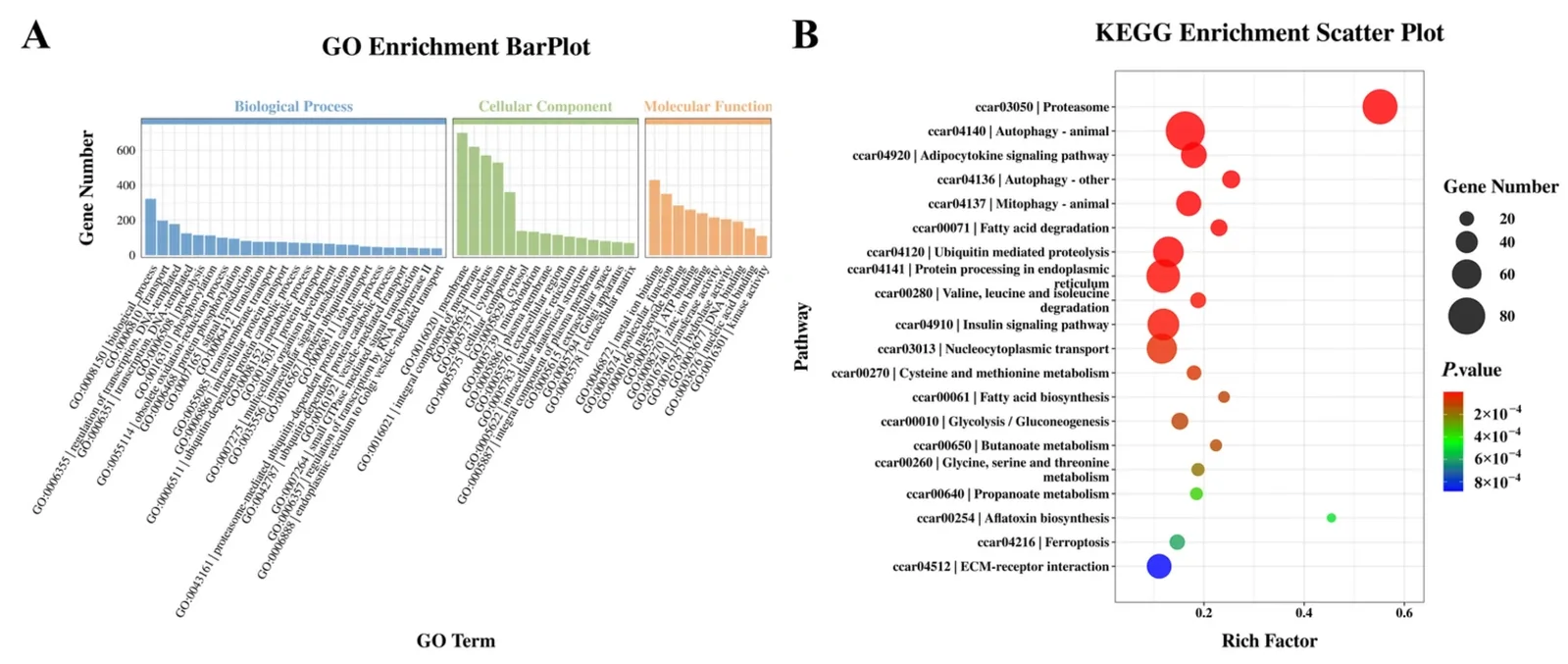
(**A**) GO enrichment analysis of significant DEGs in CJ and FR; (**B**) KEGG enrichment analysis of significant pathways in CJ and FR. The enrichment factor indicates the ratio of the number of DEGs annotated in the pathway term to the number of all genes annotated in the pathway term.

These DEGs were assigned to 3934 categories, in which we might find molecular clues explaining locomotor differences between common carp in rice fields (Table S2). GO classification indicated that genes were assigned to biological processes (such as transport, transcriptional regulation, and DNA templates), cellular components (such as the membrane, integral composition of membrane, and the content of the nucleus and cytoplasm), and molecular functions (such as metal ion binding and nucleotide binding) (Figure 2A).

KEGG enrichment analysis of the DEGs revealed potential molecular processes associated with the locomotion capacity of the common carp (Table S2). The most significantly enriched pathways included the proteasome, autophagy-animal, adipocytokine signaling pathway, and mitophagy (Figure 2B). Additionally, other key pathways, such as autophagy (other), fatty acid degradation, ubiquitin-mediated proteolysis, and protein processing in the endoplasmic reticulum, were also identified, suggesting their involvement in muscle fiber development related to locomotion regulation (Figure 2B).

### 2.2. Expression Analysis of Genes Related to Muscle Fiber Development

**Figure 3:**
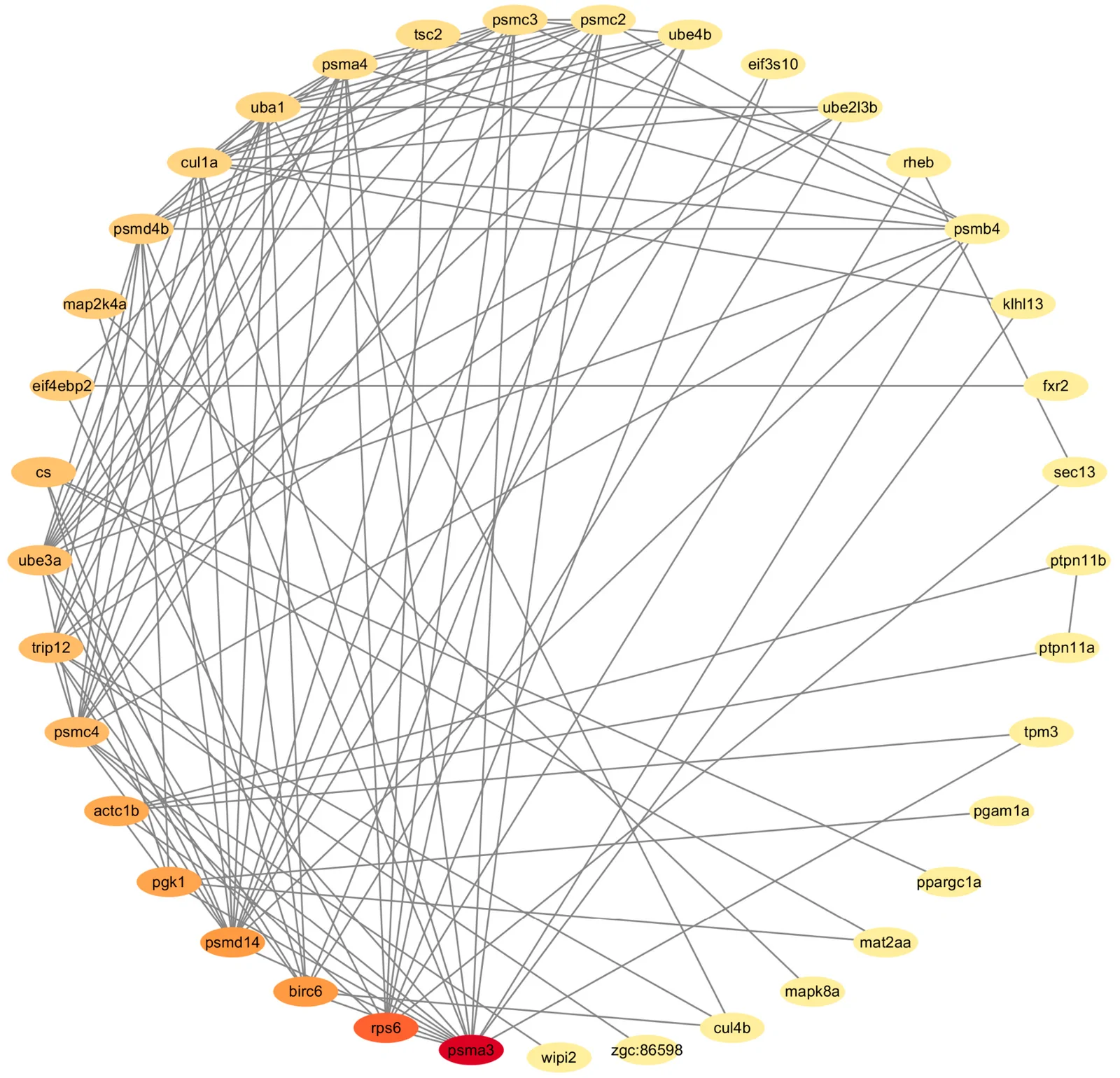
Network of the 37 co-expressed genes in the muscle.

Based on the KEGG enrichment results, 12 significantly different pathways were found between the two strains, and 52 significantly differentially expressed genes were found within these pathways (Table S3). By constructing a PPI network, the functions and interrelationships of DEGs in these 12 pathways were predicted (Figure 3). Comprehensive analysis showed 37 co-expressed genes (Table S3); among them, 14 genes (*psma*3, *psma*4, *psmc*2, etc.) associated with the ubiquitin-proteasome pathway were all upregulated in CJ. This indicated that the ubiquitin-proteasome pathway was closely related to the differential locomotion in common carp. At the same time, we also identified genes related to autophagy (*zgc*:86598 and *wipi*2), muscle contraction (*actc*1*b*, *actc*1*c*, *camk*2*b*1, *atp*1*b*1*a*, and *tpm*3), glycolysis (*ppgam*1), mitochondrial metabolism (*ppk*1) and lipid metabolism (*ptpn*11*a*, *ptpn*11*b*, *ppargc*1*a*).

### 2.3. General Information of Protein Identification

**Figure 4:**
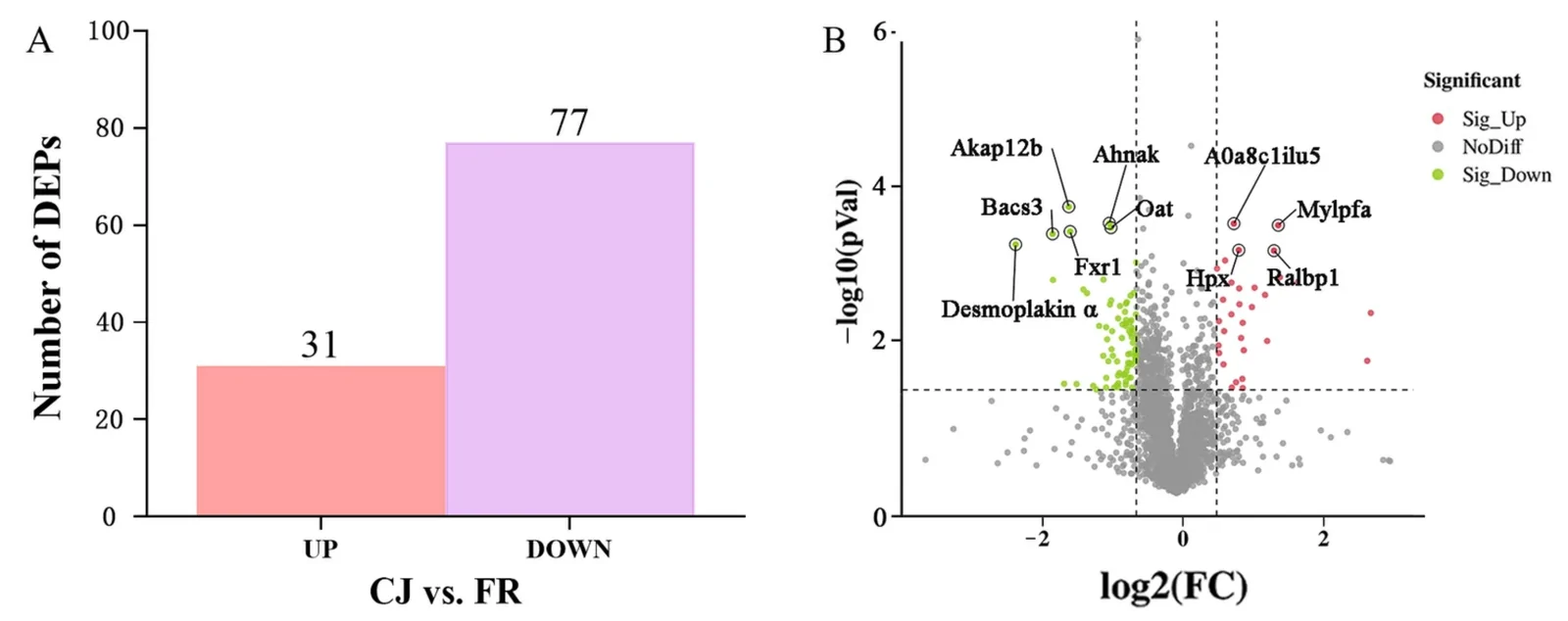
Expression analysis and quantitative comparison of differentially expressed proteins (DEPs) in the comparison of CJ versus FR. (**A**) Numbers of up- and down-regulated proteins in CJ vs. FR group. (**B**) Volcano map of DEPs. Red and green spots indicate significantly up- and down-regulated proteins, respectively.

A total of 52,352 peptide spectrum matches (PSMs) were obtained after removing low-scoring spectra. Searches using the Mascot engine identified 18,982 unique peptides, and 2742 proteins were confidently identified, with 2663 quantifiable proteins across all samples (Table S4). In total, 108 DEPs were identified, comprising 31 upregulated and 77 downregulated proteins (Figure 4A, Table S5). Among the upregulated proteins, Mylpfa (myosin light chain, phosphorylatable, fast skeletal muscle) and Hpx (hemopexin) were consistently elevated in both strains. Conversely, desmoplakin A and Bcas3 (BCAS3 microtubule-associated cell migration factor) were significantly downregulated (Figure 4B).

### 2.4. Overall GO and KEGG Pathway Enrichment Analysis of DEPs

**Figure 5:**
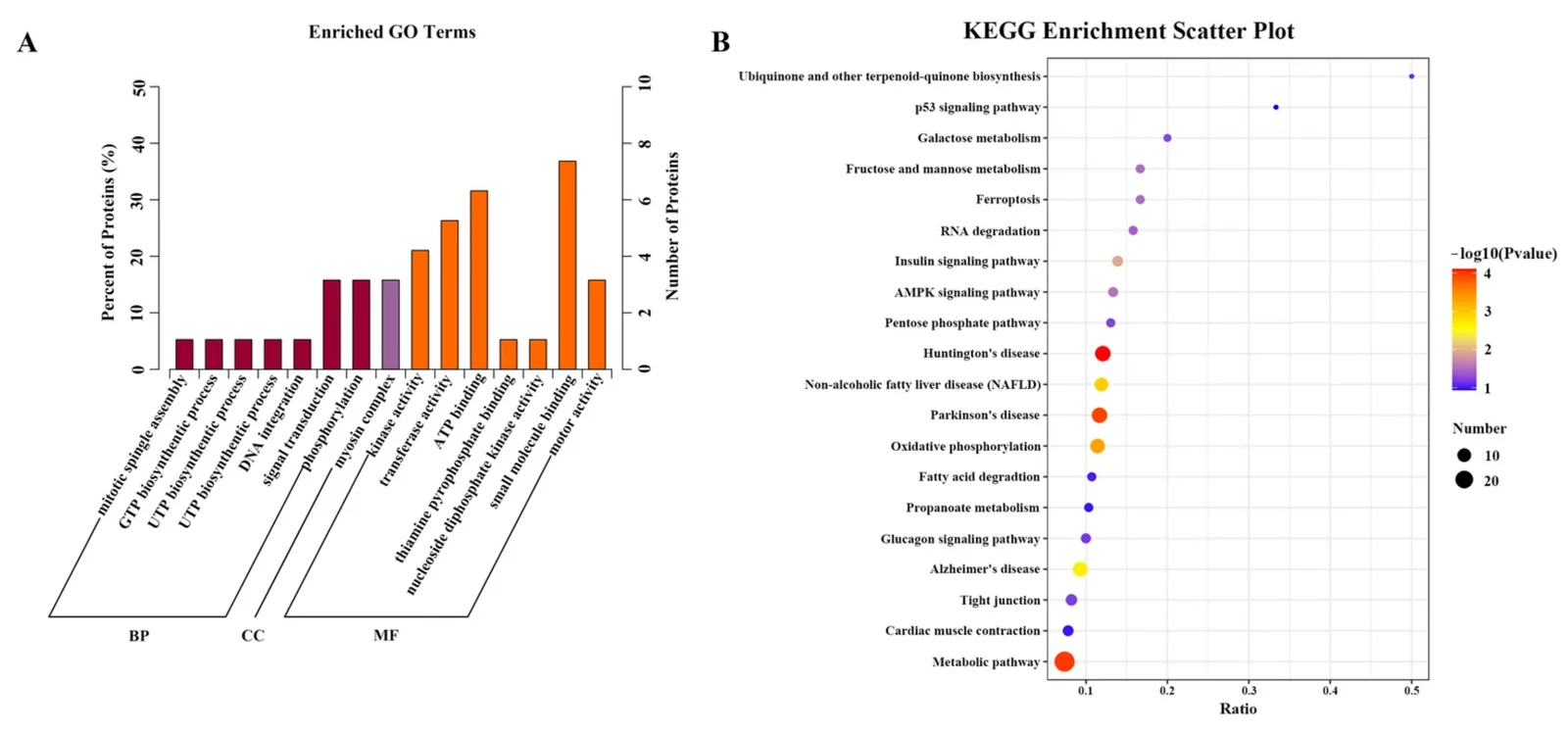
(**A**) GO enrichment analysis of significantly differentially expressed proteins in CJ and FR. (**B**) The top 20 KEGG pathway enrichment categories of significantly differentially expressed proteins. Detailed information is listed in Table S6.

The functional distribution of proteins was clarified through GO enrichment analysis. Based on the *p*-value (Figure 5A), GO enrichment showed that in biological processes, the metabolism of phosphorus-containing compounds and monomer biosynthetic process were highly enriched. For cellular components, the content of mitochondria and mitochondrial exosomes was highly enriched. In terms of molecular function, electron carrier activity and catalytic activity were highly enriched.

DEPs interact with one another in vivo, reflecting their biological activities and indicating that pathway-based annotation can facilitate our understanding of key metabolic processes related to environmental adaptation. According to the results, all identified DEPs were assigned to 80 pathways, mainly involving Huntington’s disease, metabolic pathways, and Parkinson’s disease pathways (Table S6). The bubble plot of the top 20 KEGG pathways with *p* < 0.05 was shown in Figure 5B. It was found that significantly enriched proteins were associated with pathways including oxidative phosphorylation, non-alcoholic fatty liver disease and Alzheimer’s disease.

### 2.5. Identification and Interaction Network Analysis of DEPs Related to Muscle Fiber Development

**Figure 6:**
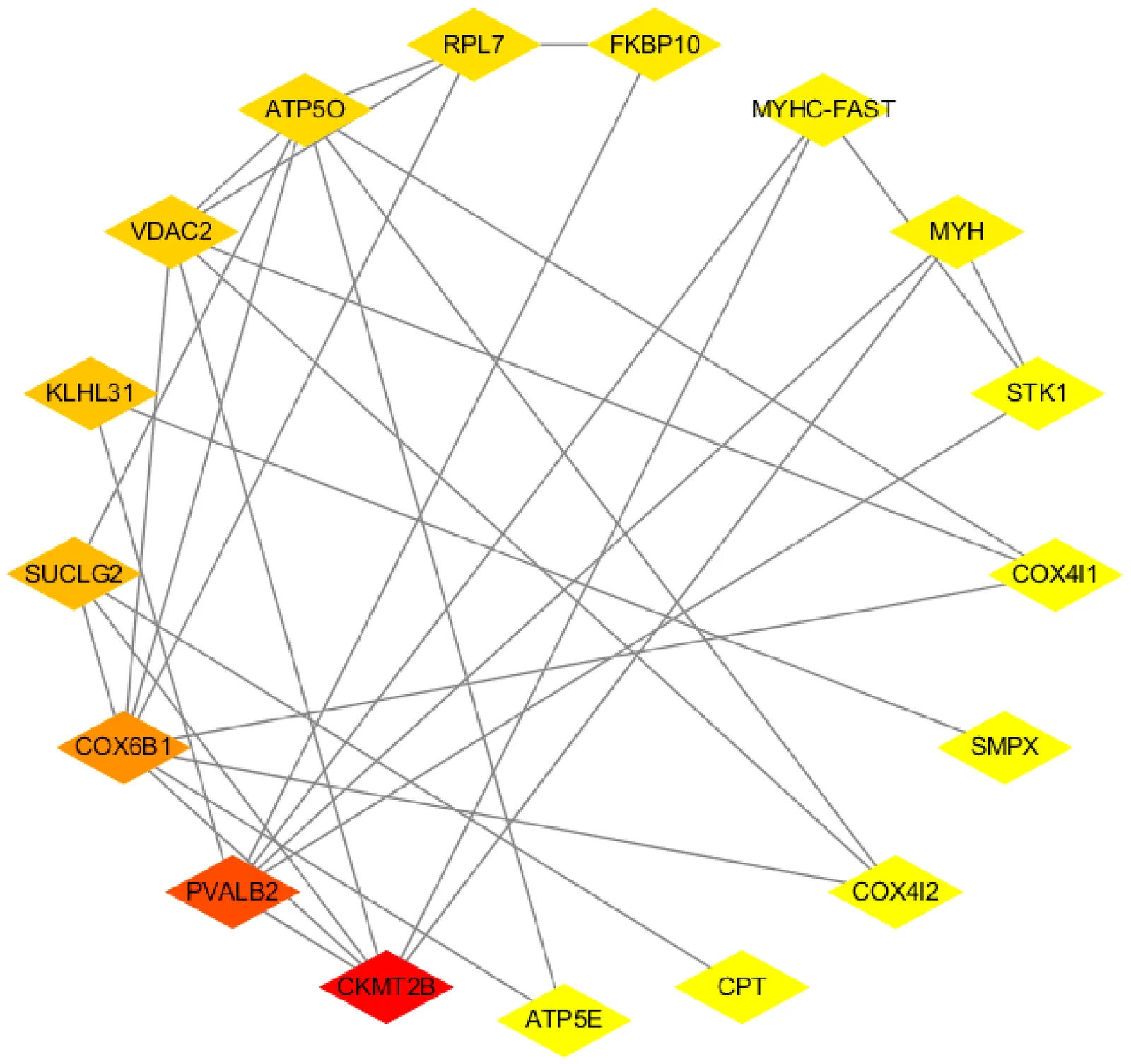
PPI network diagram of locomotion-related proteins.

A PPI network was constructed to predict the functions and interrelationships of 108 DEPs. Seventeen of these proteins overlapped with the corresponding genes identified from the transcriptomic analysis (Figure 6 and Table S7) including five up-regulated proteins, such as Myh (Myosin heavy chain embryonic type 2), Smpx (Small muscle protein) and Ckmt2b (creatine kinase, mitochondrial 2b), and 12 down-regulated proteins, such as Suclg2 (Succinate-CoA ligase subunit beta, mitochondrial), Pavlb2 (Parvalbumin-2), Atp5o (ATP synthase subunit O, mitochondrial) and Atp5e (ATP synthase subunit epsilon-like protein, mitochondrial). According to the annotations of the above proteins, most of the DEPs were involved in muscle formation (Myh and Smpx) and energy production (Suclg2, Atp5e and Atp5o), all of which are related to locomotion.

### 2.6. Integrative Analysis of Transcriptome and Proteome Data

**Figure 7:**
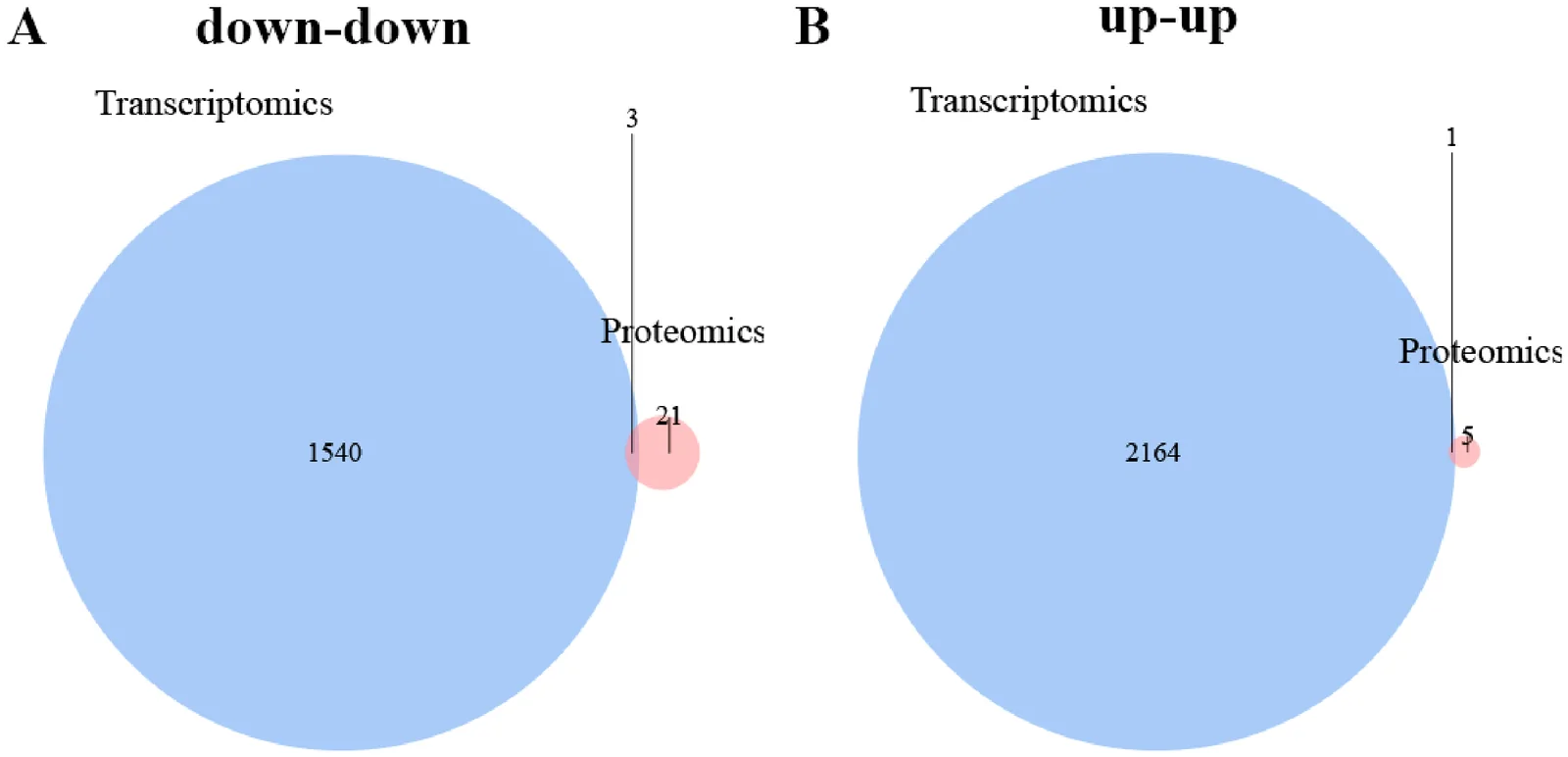
Combined analysis of transcriptome and proteome Venn diagram. (**A**) Genes and proteins up-regulated together (**B**) Genes and proteins down-regulated together.

We integrated RNA-seq and LC-MS/MS to assess the agreement between the transcriptome and proteome, as well as to understand the correlation between mRNA expression and protein abundance. Genes with significant differences in both transcription and translation levels were screened using Venn diagrams. Three overlapping mRNAs and proteins were identified as co-downregulated in CJ. They were Parvalbumin-2 (*pvalb*2), 14-3-3 protein gamma-1 (14-3-3*γ*), and peptidyl-prolyl cis-trans isomerase FKBP10 (*fkbp*10). One overlapping mRNA and protein co-upregulated in CJ was small muscle protein (*smpx*) (Figure 7 and Table S8).

### 2.7. RT-qPCR Validation of Differential Expression

**Figure 8:**
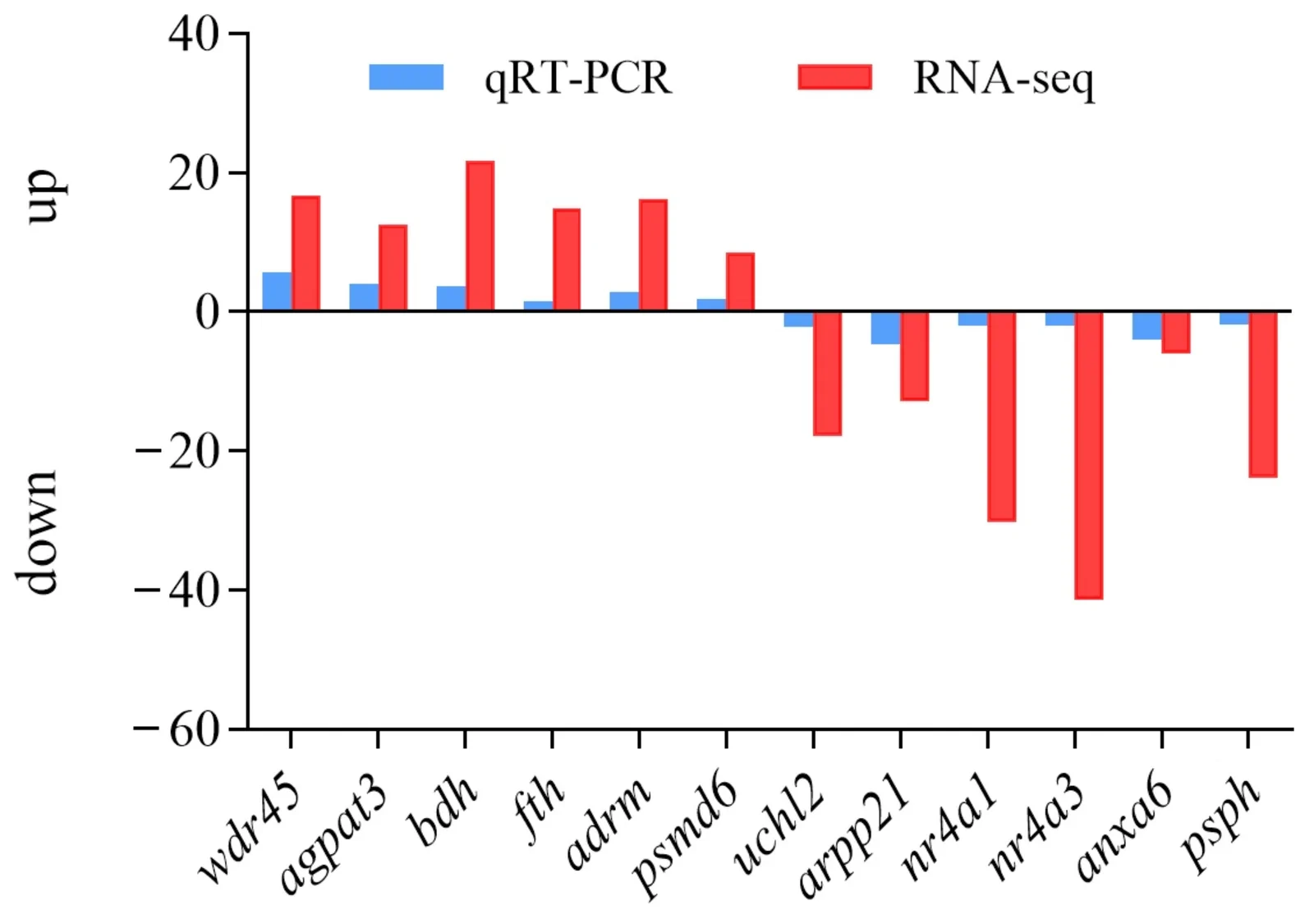
Validation of data using RT-qPCR.

To verify the reliability of transcriptome data, 12 DEGs were randomly selected for RT-qPCR analysis (Figure 8 and Table S9). It was found that the RT-qPCR expression pattern was generally consistent with those obtained by RNA-seq, indicating that transcriptome data were reliable in this study.

### 2.8. Expression of Ubiquitin-Proteasome Pathway Related Genes in CJ and FR

**Figure 9:**
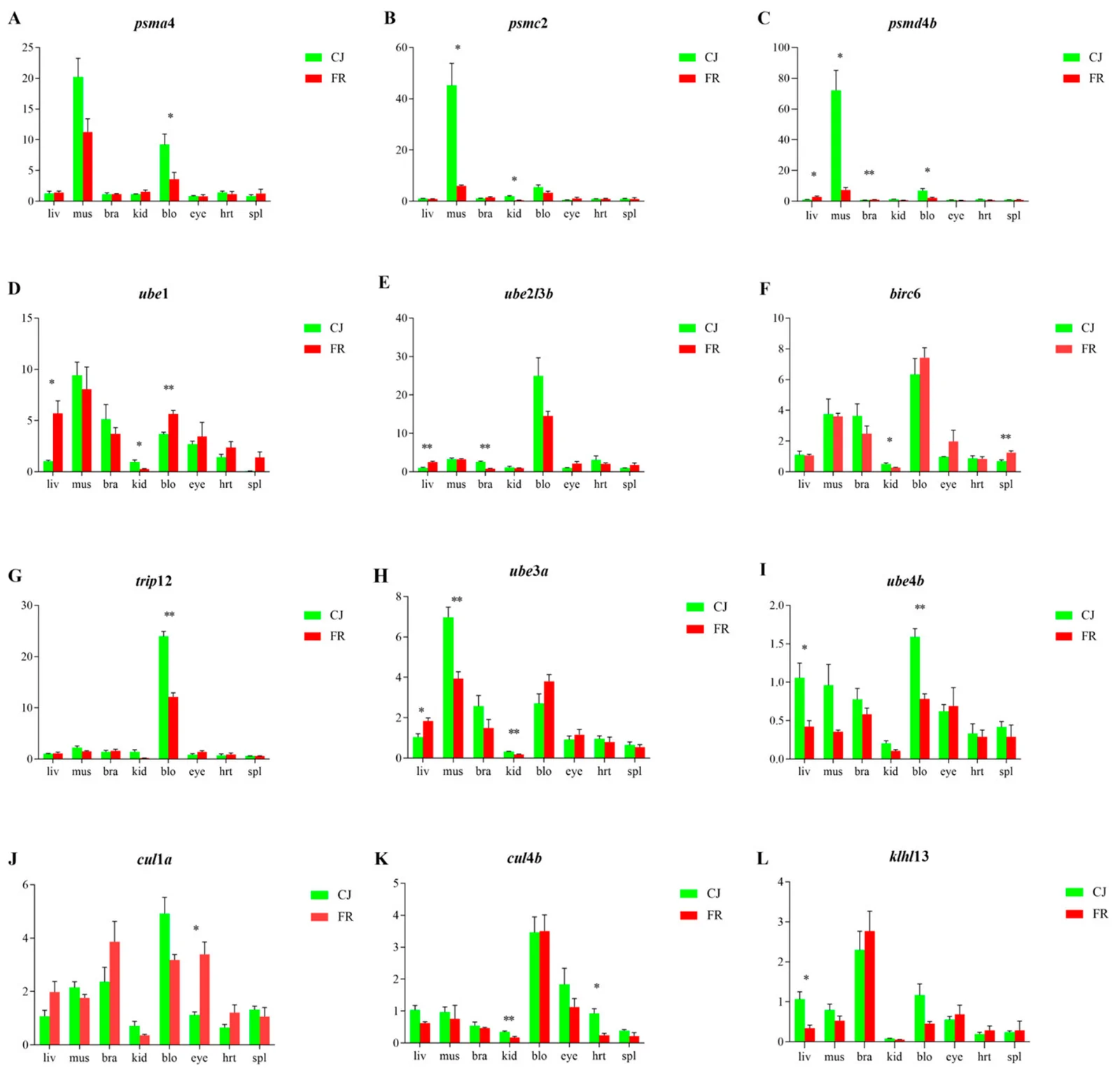
The expression of genes related to the dominant ubiquitin-proteasome pathway in different tissues of CJ and FR (proteasome: (**A**–**C**); E1: (**D**); E2: (**E**,**F**); E3: (**G**–**I**); mediated genes: (**J**–**L**). * and ** indicate significant difference at *p* < 0.05 and *p* < 0.01 levels between indicators, respectively.

Based on transcriptome enrichment results, we hypothesized that the ubiquitin-proteasome pathway may affect the locomotion of common carp. To evaluate expression levels of ubiquitin-proteasome pathway-related genes in the two common carp strains, we performed RT-qPCR detection of genes encoding proteasome subunits (*psma*4, *psmc*2, and *psmd*4*b*), E1 (*ube*1), E2 (*ube*2*l*3*b* and *birc*6), E3 (*trip*12, *ube*3*a* and *ube*4*b*) ligases, and ubiquitination-mediated genes (*cul*1*a*, *cul*4*b*, and *klhl*13) across multiple tissues of CJ and FR. The expression levels of these genes were higher in the muscle and blood of CJ relative to FR (Figure 9).

### 2.9. Effect of ube3a-dsRNA Interference on the Expression of ube3a Gene

**Figure 10:**
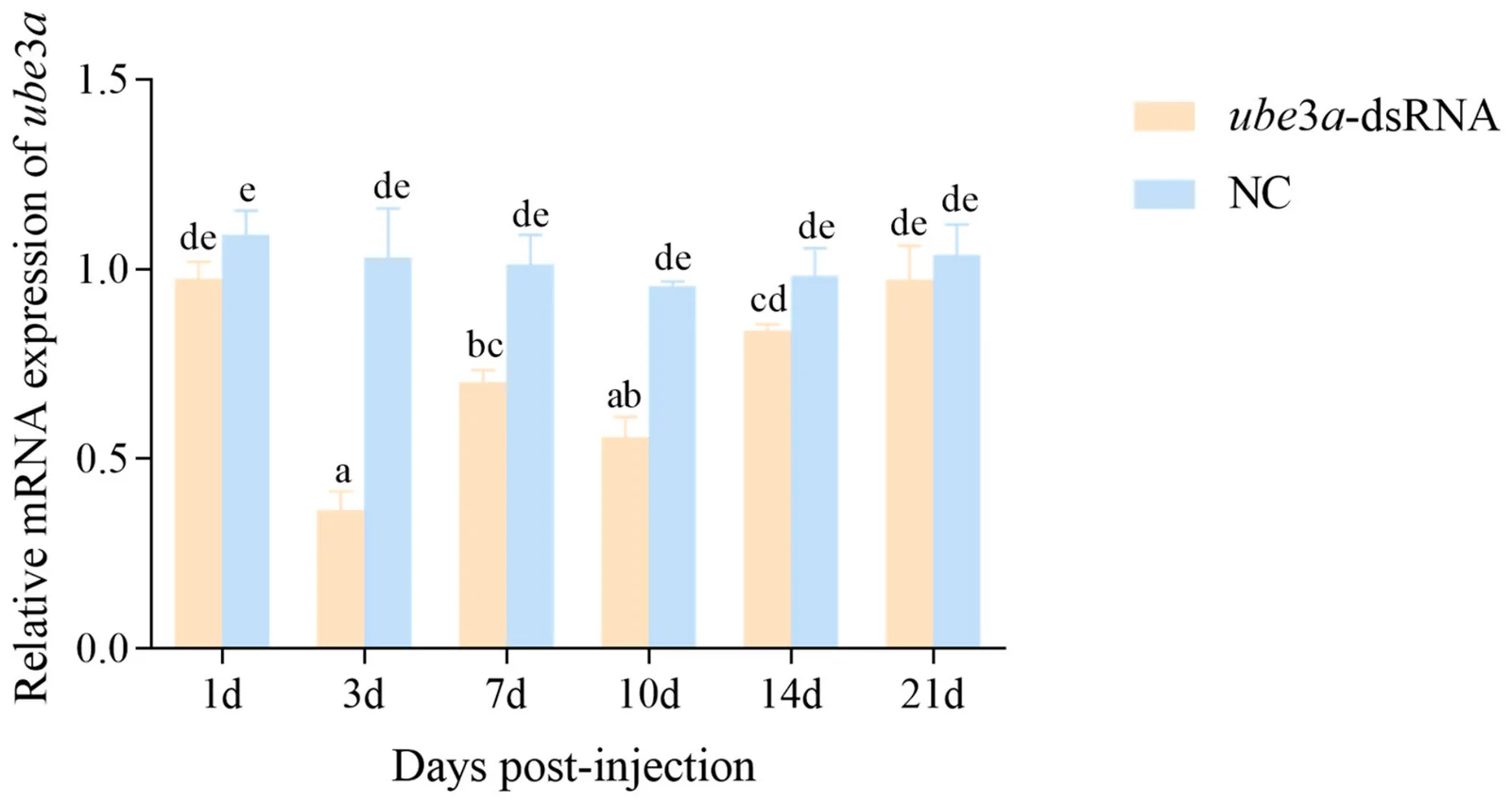
Expression analysis of *ube*3*a* mRNA in CJ muscle after injection of *ube*3*a*-dsRNA. Values without the same lowercase letter indicate significant difference (*p* < 0.05); The same as below.

RT-qPCR results showed that the *ube*3*a*-dsRNA treatment group exhibited robust gene silencing (Figure 10). The relative expression level of the *ube*3*a* gene was significantly lower than that of the control group from day 3 to day 14 post-interference (*p* < 0.05), followed by a gradual upward trend. The expression of *ube*3*a* in the experimental group reached its minimum on day 3 and increased on day 7 relative to day 3 (*p* < 0.05). The expression level decreased again on day 10 after the booster injection, and then gradually recovered to baseline levels.

### 2.10. Effect of ube3a-dsRNA Interference on the Expression of Upstream Gene (ythdc1) and Downstream Genes (stat3 and carm1) of ube3a

**Figure 11:**
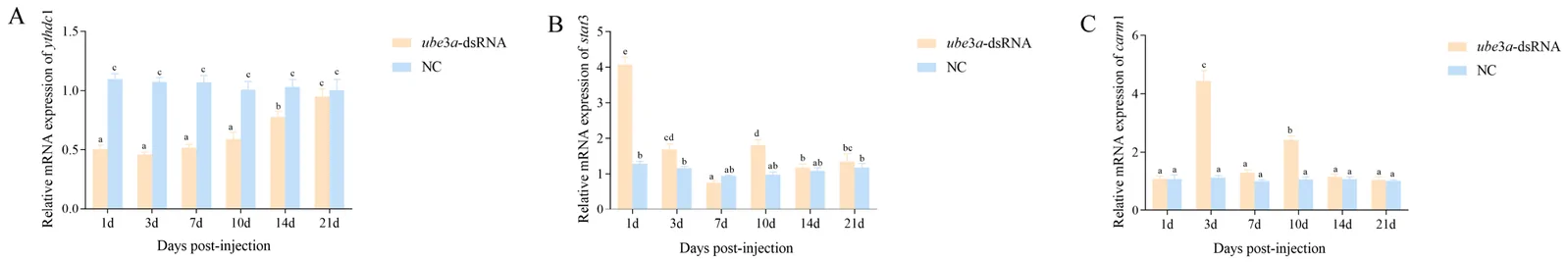
Expression analysis of *ythdc*1 (**A**), stat3 (**B**) and carm1 (**C**) mRNA in CJ muscle after injection of *ube*3*a*-dsRNA.

The expression of the upstream gene and downstream genes of *ube*3*a* following *ube*3*a* gene silencing is shown in Figure 11. The results showed that the relative expression of the *ythdc*1 gene in CJ muscle (Figure 11A) was significantly lower than that in the control group within 14 days post-interference (*p* < 0.05). No significant differences in *ythdc*1 expression were detected among days 1, 3, 7 and 10 in the experimental group (*p* > 0.05). *ythdc*1 expression in the experimental group gradually recovered to baseline levels after day 10, and no difference was observed between the experimental and control groups at day 21 (*p* > 0.05). The relative expression of the *stat*3 gene in CJ muscle (Figure 11B) in the interference group peaked at day 1 and was significantly higher than that in the control group (*p* < 0.05). After day 1, *stat*3 mRNA expression declined until returning to baseline. Following the booster injection of *ube*3*a*-dsRNA at day 7, *stat*3 mRNA expression was again significantly higher than in the control group at day 10 (*p* < 0.05), and then decreased and returned to baseline. The relative expression of the CJ *carm*1 gene in CJ muscle (Figure 11C) in the experimental group was significantly higher than that in the control group only at day 3 and day 10 post-interference (*p* < 0.05); no significant differences were found between the two groups at other time points (*p* > 0.05).

### 2.11. Effect of Injection of ube3a-dsRNA on the Expression of Ube3a in CJ Muscle

**Figure 12:**
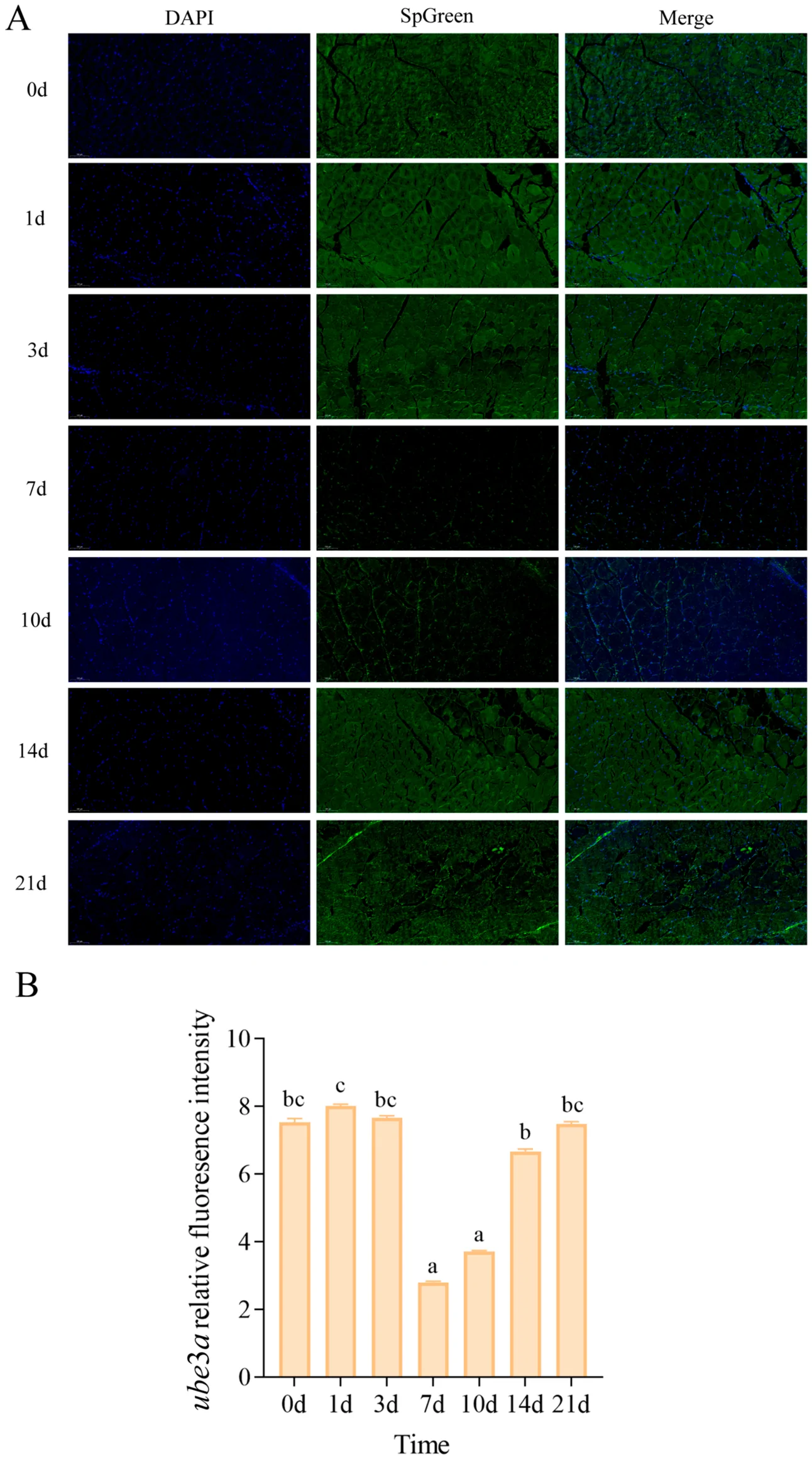
Immunofluorescence results of Ube3a from CJ muscle after injection of *ube*3*a*-dsRNA. (**A**). Immunofluorescence detection of muscle tissue post-interference of the *ube*3*a* gene. (**B**). Comparison of relative fluorescence intensity of Ube3a between muscle tissues after *ube*3*a* interference.

**Figure 13:**
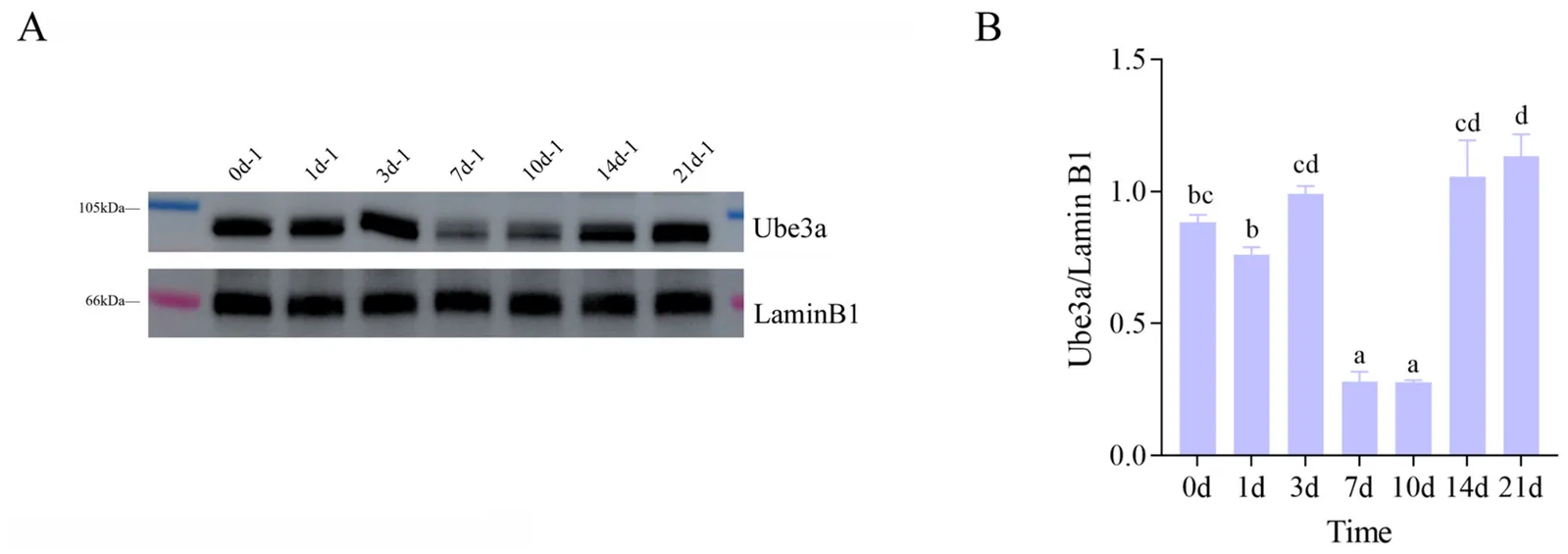
Western blotting results of Ube3a protein in CJ muscle after injection of *ube*3*a*-dsRNA. (**A**). Changes in protein expression levels in muscle tissue after *ube*3*a* interference. (**B**). Comparison of protein grayscale values in muscle tissue after *ube*3*a* interference.

Immunofluorescence and western blotting were used to compare Ube3a protein abundance between the interference and control groups. Immunofluorescence results showed that Ube3a protein levels in CJ muscle reached a minimum at days 7 and 10 relative to other time points after *ube*3*a*-dsRNA injection (*p* < 0.05) (Figure 12A,B). The WB results showed the same trend as the fluorescence quantification results. (Figure 13A,B).

### 2.12. The Effect of ube3a-dsRNA Interference on Muscle Fibers of CJ

**Figure 14:**
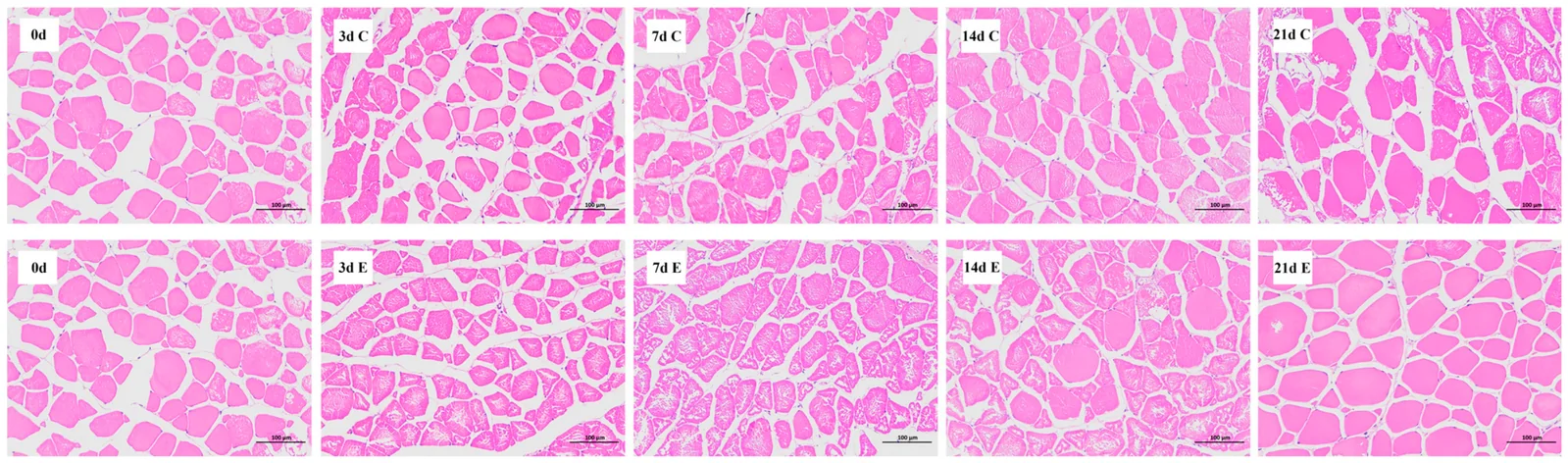
Transverse sections of CJ muscle fibers showing the fiber diameter and fiber density differences between the interference group (E) and control group (C) at various time points after *ube3a* dsRNA injection.

**Figure 15:**
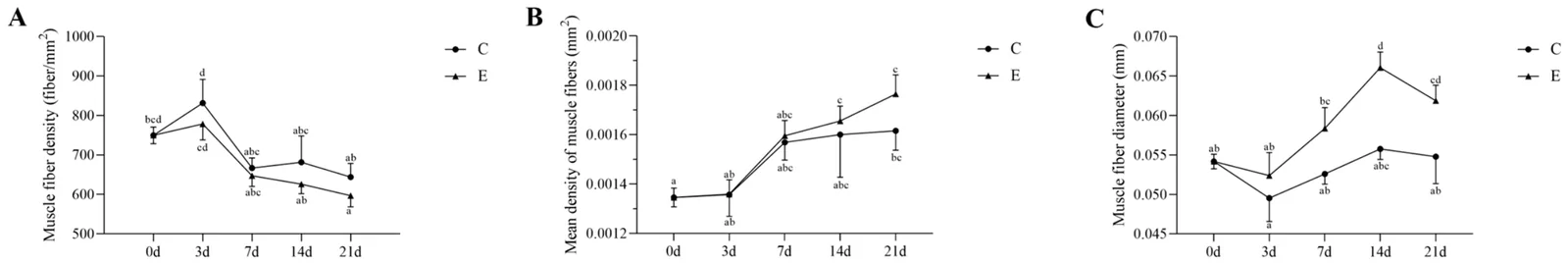
Characteristics of CJ muscle fibers between the interference group (E) and control group (C) after injection of *ube*3*a*-dsRNA ((**A**): muscle fiber density; (**B**): mean area of muscle fibers; (**C**): muscle fiber diameter).

Histological sections of muscle tissue revealed differences in muscle fiber characteristics between the interference and control groups starting at day 14 after *ube*3*a*-dsRNA injection (Figure 14). Specifically, following *ube*3a-dsRNA injection, muscle fiber density in the CJ experimental group was consistently lower than that in the control group. From day 0 to day 7, the difference in muscle fiber density between the experimental and control groups first increased and then decreased (Figure 15A). After the booster injection on day 7, the difference in fiber density increased again and subsequently declined. Muscle fiber density at day 21 was significantly lower than that at day 0 (*p* < 0.05). Altered muscle fiber morphology, consistent with hypertrophy, was observed. The average muscle fiber area in the experimental group was larger than that in the control group from day 0 to day 21, and the difference between groups increased over time (Figure 15B). The average muscle fiber area was significantly greater at day 14 than that at day 0 (*p* < 0.05), and significantly higher at day 21 than that at day 0 (*p* < 0.01). Additionally, the difference in muscle fiber diameter between the experimental and control groups continuously increased from day 3 to day 14 (Figure 15C). Muscle fiber diameter was significantly higher at day 14 (*p* < 0.01) and day 21 (*p* < 0.05) compared with day 0.

## 3. Discussion

The retention of fish cultured in paddy fields significantly affects the harvest of food fish in the rice-fish symbiosis systems. Fish retention in paddy fields is correlated with their locomotor capacity; greater active movement may cause higher escape risk. In our previous research, we evaluated the muscle characteristics of two common carp strains, the sluggish Congjiang strain (CJ) and active FFRC No. 2 strain (FR), cultured in a paddy field belonging to a GIAHS in Guizhou Province. We found that muscle characteristics (nutritional composition, muscle fiber and muscle texture) affected fish locomotor capacity [5]. According to the defined threshold, 3708 DEGs and 108 DEPs were identified, and GO enrichment and KEGG enrichment analyses were performed simultaneously to better understand the complex metabolic network correlated with the target trait. Based on the DEGs within the top-ranked KEGG pathway, a PPI network was constructed to predict the functions and interrelationships of DEGs in the pathway. It was found that a total of 37 genes (including *psmc*2, *ptpn*11*a*, *ppargc*1*a* and *psmc*4) were co-expressed.

Earlier researchers reported that in teleosts, skeletal muscle growth relies on the hypertrophic growth of muscle fibers [11]. Muscle fiber hypertrophy relies on two mechanisms: (1) protein deposition within mature muscle fibers, and (2) myogenic stem cells are activated, proliferate, differentiate into myoblasts, and then fuse with existing mature muscle fibers to promote muscle fiber hypertrophy [12]. In our previous research, it was found that the diameter and area of CJ muscle fibers were smaller than those of FR muscle fibers [5]. This phenomenon might result from reduced protein deposition and cell fusion in CJ muscle, or enhanced protein degradation in CJ muscle. Interestingly, the transcriptome PPI network contained 14 genes associated with the ubiquitin-proteasome pathway. As a major protein degradation pathway, the ubiquitin-proteasome pathway (UPP) mediates the selective degradation of proteins in eukaryotic cells.

The genes related to the ubiquitin-proteasome pathway have been extensively studied in humans and mice [13,14], but remain poorly characterized in fish. In this study, three proteasomes (*psma*4, *psmc*2 and *psmd*4*b*) exhibited significantly different expression levels in the muscles between CJ and FR. As the primary protein degradation system in eukaryotic cells, the UPP mediates selective degradation of target proteins through the 26S proteasome. This process involves the coordinated action of ubiquitin (UB), ubiquitin-activating enzyme (E1), ubiquitin-conjugating enzyme (E2), ubiquitin ligase (E3), and deubiquitinating enzymes (DUBs) [15,16]. In the ubiquitin-proteasome pathway, E1 is responsible for initiating the entire protein ubiquitination process. During the ubiquitin-proteasome pathway, E3 ligases have a specific recognition function for target proteins. In this study, three E3 ligases, namely *ube*3*a*, *ube*4*b* and *trip*12, showed higher expression in CJ relative to FR. T*rip*12 was found to recognize and degrade SOX6 in mice, inducing changes in fiber type-specific gene expression in muscle cells [17]. Ube3a was found to affect the expression of parvalbumin in mice [18]. In this study, the gene and protein Parvalbumin were identified through transcriptome and proteomic analysis, indicating that *ube*3*a* may be a key gene leading to muscle fiber differences between CJ and FR. To investigate the function of *ube*3*a* on carp muscles, we performed a 21-day in vivo *ube*3*a* knockdown experiment. The interference results showed that following *ube*3*a*-dsRNA injection, *ube*3*a* mRNA expression in CJ reached its minimum on day 3, whereas both WB and IF data demonstrated that Ube3a protein abundance was significantly lower on days 7 and 10 compared with other time points. Although mRNA abundance significantly decreased after interference, the proteins often possess longer half-lives, or cells maintain protein homeostasis by boosting translation efficiency [19]. Based on the HE staining results, the muscle fiber density in the experimental group was lower than that in the control group, while the average muscle fiber area and diameter were higher than those in the control group. These results suggested that *ube*3*a* exerts an inhibitory effect on the muscle development of common carp. However, in the absence of swimming performance assays, we cannot conclude that *ube*3*a* directly regulates locomotor capacity. Direct measurement of swimming performance represents a promising follow-up for this study. *ube*4*b* shares functional similarities with *ube*3*a* [20,21]. Moreover, three ubiquitination-mediating scaffold genes, *cul*4*b*, *cul*1*a* and *klhl*13 [22], were also differentially expressed between CJ and FR in this study. Sweich et al. [23] reported that high expression of *cul*4*b* promoted the ubiquitination and degradation of TSC2, and negatively regulated mTOR signaling. The rapamycin-sensitive mTORC1 regulates various intracellular processes, including translation, autophagy, cell cycle and microtubule dynamics [24]. Based on previous research, we speculate that elevated *cul*4*b* expression in CJ muscle may promote Tsc2 ubiquitination and degradation, thereby suppressing mTORC1 activity, ultimately reducing protein translation in CJ muscle cells and inhibiting muscle fiber hypertrophic growth. In addition, *cul*1*a* has similar functions to *cul*4*b*, and its mechanism of action in muscle cells might be similar.

Discordance between mRNA and protein abundance is a common biological phenomenon, which can be shaped by translational regulation, protein stability, post-translational modifications and other regulatory mechanisms. Zhu et al. [25] applied distinct filtering thresholds for transcriptome and proteome data in their study on grass carp growth. Accordingly, the findings of this study provide candidate pathways, differentially expressed genes and proteins for further validation, rather than definitive conclusions. From the proteomic dataset, we identified that Klhl31 and Klhl13, two members of the Kelch family, were differentially abundant between the two strains. KLHL31 and KLHL13 were both found to be involved in ubiquitination [26,27]. Meanwhile, Abou Elhamd et al. [28] found that the effect of KLHL31 on sarcomere and sarcomere size was mediated by inhibiting Wnt signaling, and knocking down KLHL31 increased cell proliferation and myogenesis. Based on previous research, we speculate that high Klhl31 and Klhl13 levels might promote protein degradation in the muscle of CJ.

In recent years, multiple studies have revealed a significant crosstalk between the ubiquitin-proteasome system and autophagy [29,30]. One mechanism of communication is that changes in one proteolytic pathway may induce changes in another pathway. Another communication mechanism is that a component of one degradative pathway regulates a subunit, or becomes a substrate, of another pathway. In the transcriptome PPI network, we detected autophagy-related DEGs (*zgc*: 86598 and *wipi*2), which might interact with genes related to the ubiquitin-proteasome system. Geisler et al. [31] found that *ube*2*l*3 synergistically promoted Parkin-mediated mitochondrial autophagy with *ube*2*n* and *ube*2*d*2/3. Furthermore, knockdown of the E2 enzymes *ube*2*n*, *ube*2*l*3 or *ube*2*d*2 and *ube*2*d*3 (*ube*2*d*2/3) significantly reduced autophagic clearance of depolarized mitochondria. In this study, *zgc*: 86598 and *ube*2*l*3*b* were both upregulated in CJ muscle. Based on previous research, we speculate that the interaction between *zgc*: 86598 and *ube*2*l*3*b* in common carp might belong to the first mechanism of communication between UPP and autophagy, i.e., the expression of *ube*2*l*3*b* affected the expression of *zgc*: 86598. Wan and Liu [32] reported that WIPI2 acts as a phosphorylation substrate of mTORC1 and a ubiquitination target of E3 ubiquitin ligase HUWE1. In this study, the interaction between *wipi*2 and two other ubiquitin ligase E3 (*ube*3*a* and *trip*12) in muscle might be similar to its interaction with *huwe*1. This interaction belonged to the second communication mechanism between UPP and autophagy, where *wipi*2 participated in the autophagy process and might also be a target for ubiquitination by *ube*3*a* and *trip*12. This hypothesis requires validation via future functional assays. It was also worth noting that *birc*6 negatively regulated autophagy in mice [33]. Both *birc*6 and *ube*2*l*3 belong to E2-binding enzymes, which are involved in ubiquitination and autophagy. We speculated that E2-binding enzymes played a role in connecting UPP and autophagy in fish, which awaits verification through further functional experiments.

In this study, some genes (*pgam*1, *pgk*1, *ptpn*11*a*, *ptpn*11*b*, *ppargc*1*a* and *mat*2*aa*) and proteins (Cox7b1, Cox4i1 and Cox4i2) related to energy metabolism were down-regulated in CJ. PGAM1 knockdown in HaCaT cells significantly inhibited the LPA-induced cell proliferation and expression of cell cycle regulators [34]. PPARGC1A facilitated mitochondrial biogenesis and regulated skeletal muscle metabolism by mediating glycolysis and the tricarboxylic acid (TCA) cycle. Furthermore, *ppargc*1*a* promoted intramuscular fatty acid oxidation, drove the transformation of fast-twitch to slow-twitch myofibers, and increased skeletal muscle mass [35]. The expression of these energy-metabolism-associated genes and proteins was lower in CJ muscle. We speculated that CJ might produce less energy, leading to reduced locomotor capacity. This result is consistent with our previous finding of lower ROS content in CJ muscle [5]. However, whether the low expression of energy metabolism genes in CJ is directly linked to reduced swimming capacity needs further validation with swimming performance measurements.

Overall, the expression of ubiquitin-proteasome-related genes in different tissues of fish is similar to that in mice, indicating that the regulatory mechanism of the ubiquitin-proteasome pathway in fish may be similar to that in mice. Importantly, the omics differences observed in this study reflect the integrated phenotypic divergence between the two strains under specific culture conditions and thus cannot be attributed solely to locomotor behavior. These genes were all upregulated in CJ muscle; we speculated that they might promote protein degradation via enhanced ubiquitination catalysis and thereby suppress hypertrophic growth of muscle fibers. Our in vivo *ube*3*a* knockdown assay in CJ further demonstrated that the ubiquitin-proteasome pathway can regulate muscle fiber development in common carp. In addition, multiple genes involved in energy metabolism processes (*pgam*1, *pgk*1, *ppargc*1*a*, etc.) and their encoded proteins (Cox7b1, Cox4i1, Cox4i2, etc.) were downregulated in CJ. We speculate that impaired energy production results in the reduced locomotor capacity of CJ. These hypotheses can be addressed in future work through whole-genome resequencing, promoter SNP analysis and eQTL analysis.

## 4. Materials and Methods

### 4.1. Sample Collection for Transcriptome, Proteome and qRT-PCR Analysis

Three 1-year-old Congjiang strain common carp (CJ, approximately 200 g) individuals and FFRC No. 2 strain common carp (FR, approximately 500 g) individuals were randomly selected from the Taijiang Experimental Base of Guizhou Fisheries Research Institute, respectively. Individuals from both strains were one year old, but their growth rates differed because of inherent strain differences. Muscle (mus), liver (liv), brain (bra), kidney (kid), blood (blo), eye (eye), heart (hrt), and spleen (spl) tissues were collected immediately after sacrifice. All tissues were rapidly frozen in liquid nitrogen and then stored at −80 °C for subsequent analysis. A portion of the muscle tissue was allocated for transcriptomic and proteomic sequencing; another portion of the muscle tissue, along with other collected tissues, was preserved for RT-qPCR detection.

### 4.2. Sequencing of the Transcriptome

Three CJ and three FR muscle tissues were used for transcriptome analysis. Total RNA was extracted from muscle tissues using TRIzol reagent (Invitrogen, CA, USA). RNA library construction required the following quality parameters: RNA concentration ≥ 35 ng/μL, total RNA amount ≥ 1 μg, OD 260/230 ratio ≥ 1, and OD 260/280 ratio ≥ 1.8. Reverse transcription and sequencing were performed on the Illumina Novaseq 6000 platform (Illumina, Inc., San Diego, CA, USA).

Sequence alignment was conducted using TopHat2v2.0.13 (Center for Computational Biology, University of Maryland, College Park, MD, USA; (http://tophat.cbcb.umd.edu/ (accessed on 20 September 2022)), followed by transcript assembly and splicing using Cufflinks software v2.2.1 (Cole Trapnell Laboratory, University of Washington, Seattle, WA; (https://cole-trapnell-lab.github.io/cufflinks/manual/ (accessed on 20 September 2022)) to obtain transcripts for subsequent functional annotation. The genome alignment results and RSEM genome annotation files were used to acquire transcript read counts [36], with gene expression levels quantified using FPKM or TPM normalization. Differential expression analysis between groups was implemented with DESeq2 based on the negative binomial distribution model. The Benjamini-Hochberg procedure was applied for multiple-testing correction to obtain the false discovery rate (FDR).

Differentially expressed genes (DEGs) were identified with the thresholds of |log_2_(FC)| > =1 and *q* (FDR) < 0.05. Fold change was defined with the thresholds of FC >= 2 or FC <= 0.5. Functional enrichment analysis of DEGs, including Gene Ontology (GO) and KEGG pathway enrichment, was performed using Goatools and KOBAS, respectively. Based on our previous research methodology [37], protein-protein interaction (PPI) network analysis was conducted to generate network diagrams and elucidate relationships among key DEGs. Betweenness centrality was calculated using the CytoNCA plugin v2.1 (East China Normal University, Shanghai, China) in Cytoscape v3.7.1 (Cytoscape Consortium, San Diego, CA, USA) and mapped to a node color gradient to identify critical bridge nodes within the PPI network.

### 4.3. Sequencing of the Proteome

The muscle proteomic analysis was performed according to previously published methods [37]. Total proteins were extracted from muscle tissue using urea lysis buffer containing protease inhibitors. For protein digestion, 100 μg of protein was reduced with TCEP [tris(2-carboxyethyl)phosphine] at 37 °C for 60 min, followed by alkylation with 40 mM iodoacetamide in the dark at room temperature for 40 min. Proteins were then precipitated with acetone at −20 °C for 4 h and centrifuged at 10,000× *g* for 20 min before tryptic digestion overnight at 37 °C. For tandem mass tags (TMT) labeling, the TMT reagent (Cat. No. A44522, Thermo Fisher Scientific, Waltham, MA, USA) was dissolved in acetonitrile and added to peptides; the solution was incubated for 2 h at room temperature, followed by quenching with hydroxylamine for 30 min. The labeled samples were pooled in equal amounts and dried using a vacuum concentrator. Liquid chromatography-tandem mass spectrometry (LC-MS/MS) analysis was performed using high pH reverse-phase separation for deeper proteome coverage. Peptides were reconstituted in UPLC loading buffer (2% acetonitrile) and separated on a C18 column, followed by two-dimensional LC-MS/MS analysis according to standard protocols from Majorbio Biomedical Technology Co., Ltd. (Shanghai, China). Raw data were processed using Proteome Discoverer software (Thermo Fisher, version 2.4) with mass tolerances of 20 ppm for precursors and 0.02 Da for fragments, requiring at least one unique peptide for protein identification. Data analysis was performed using the free online platform (cloud.majorbio.com accsessed on 27 September 2022). Differentially expressed proteins (DEPs) were identified with the thresholds of |log_2_(FC)| >= 0.585 and *p* < 0.05. Fold change was defined with the thresholds of FC >= 1.5 or FC <= 0.67, followed by GO (http://geneontology.org/ (accessed on 27 September 2022)) and KEGG (http://www.genome.jp/kegg/ (accessed on 27 September 2022)) enrichment analyses. Protein-protein interaction (PPI) networks were constructed using STRING v12.0 (SIB Swiss Institute of Bioinformatics, Lausanne, Switzerland; https://string-db.org (accessed on 25 October 2024)) software and visualized with Cytoscape v3.7.1 (Cytoscape Consortium, San Diego, CA, USA). The results of proteomic screening are considered candidate results, rather than direct conclusions.

### 4.4. Combined Analysis in Transcriptomics and Proteomics

Before conducting the combined analysis, we screened differentially expressed genes and proteins. For mRNAs, differentially expressed genes (DEGs) were identified with the thresholds of |log_2_(FC)| > =1 and *q* < 0.05. Fold change was defined with the thresholds of FC >= 2 or FC <= 0.5. For proteins, differentially expressed proteins (DEPs) were identified with the thresholds of |log_2_(FC)| >= 0.263 and *p* < 0.05. Fold change was defined with the thresholds of FC >= 1.2 or FC <= 0.83. Firstly, by taking the intersection of Venn diagrams, genes with significant differences at both transcription and translation levels were screened. Secondly, the relevant information of the intersection genes was listed for each group. Finally, GO annotation and enrichment analysis, as well as KEGG pathway analysis, were performed on all interactions between mRNAs and proteins.

### 4.5. Quantitative PCR Analysis

In order to verify the accuracy of the sequencing results, we randomly selected 12 candidate genes with different expression levels for real-time fluorescence quantitative PCR validation, including 6 up-regulated genes and 6 down-regulated genes (Table S9). In addition, we also analyzed the expression of ubiquitin-proteasome pathway-related genes in different tissues of CJ and FR. The primers for 25 genes are shown in Table S9. Primer specificity was confirmed by single melting peaks on melting curve analysis. The relative quantification of the target and reference genes was evaluated according to standard curves. Each experiment was conducted four times. Referring to other studies on common carp muscle [38], we chose *β-actin* as the internal reference gene. Total RNA was extracted from the test sample using Trizol reagent (ThermoFisher, 15596018) according to the manufacturer’s procedure. Afterward, cDNA was synthesized using the PrimeScript ™ the RT reagent kit with gDNA Eraser (Perfect Real Time) (RR047A, TakaraBio Inc., Kusatsu, Shiga, Japan) kit. Real-time fluorescence quantitative PCR was performed following our previous research [39] on a CFX96 Real-Time PCR System (Bio-Rad, Hercules, CA, USA). The reaction system was 10 μL, consisting of 5 μL 2 × SYBR Green Master Mix, 1 μL cDNA template, 0.2 μL (10 μmol/L) upstream primer, 0.2 μL (10 μmol/L) downstream primer, and 3.6 μL H_2_O. The reaction was run based on the following protocol: denaturing at 95 °C for 5 min, followed by 40 cycles of denaturing at 95 °C for 15 s and then annealing at 60 °C for 45 s.

### 4.6. Preparation of CJ ube3a-dsRNA

Based on the conserved sequence of *ube*3*a*, specific primers *dsube*3*a*-1F/*dsube*3*a*-1R and *dsube*3*a*-2F/*dsube*3*a*-2R were designed using the online tool SnapDragon dsRNA Design (http://www.flyrnai.org/cgi-bin/RNAi_find_primers.pl (accessed on 26 May 2025)) for double-stranded RNA preparation. The T7 promoter sequence (TAATACGACTCACTATAGGGAGA) was added to the 5′ end of both forward and reverse primers. All primers were synthesized by Biotechnology (Shanghai) Co., Ltd.; Shanghai, China, and their sequences are listed in Table 1.

Using cDNA synthesized from the RNA extracted from the dorsal muscle tissue of CJ as the template, PCR amplification was carried out with a 2× Taq PCR Master Mix kit (Conway Reagent, Taizhou, Jiangsu, China). Using purified PCR products as templates, dsRNA corresponding to the *ube*3*a*-1 and *ube*3*a*-2 transcripts was synthesized in vitro using the MEGA Script RNAi Kit (20 RXN; Invitrogen, Shanghai, China) according to the manufacturer’s instructions. Subsequently, the quality and concentration of the dsRNA were assessed, and an appropriate volume of Elution Solution was added to dilute the product to the working concentration required for the experiment. The prepared dsRNA was aliquoted and stored at −20 °C until use.

### 4.7. Time-Dependent Interference Trial

CJ juvenile fish weighing 8.3 ± 2.1 g were selected from the Taijiang Experimental Base of the Guizhou Fisheries Research Institute as experimental fish in interference trials. After one week of acclimation in a recirculating aquaculture system, the fish were randomly allocated into two groups. Each group had three replicate tanks, with 8 fish per tank. The trials were conducted under controlled conditions: temperature was maintained at 28 ± 2 °C, and a 12-h light/dark cycle was applied. Compound aquatic feed was provided daily at fixed intervals in the morning and evening. Fish were anesthetized in buffered tricaine methane sulfonate (MS-222, 80–100 mg L^−1^; pH adjusted to 7.0–7.5 with sodium bicarbonate) prior to injection and tissue sampling. Fish were returned to aerated fresh water for recovery after manipulation. The two groups were designated as follows: the experimental group (E), injected with *ube*3*a*-dsRNA, and the blank control group (C), injected with an equal volume of physiological saline. Based on the protocol established in common carp by Song et al. [9], an injection dose of 5 μg/g was administered intramuscularly using a high-temperature sterilized microsyringe. To prevent leakage of the injected solution, the needle was retained in the fish for 30 s after injection. The initial injection was performed on day 0 (0 d), followed by a booster injection on 7 d (at the same concentration as the first injection, 5 μg/g). The control group received an equal volume of saline solution using the same procedure. Mortality in each group was recorded throughout the experiment. Muscle samples were collected from the dorsal region of CJ fish before injection (0 d) and on 1 d, 3 d, 7 d, 10 d, 14 d, and 21 d post-injection (Table S10). At each time point, six fish per group (two from each replicate tank) were sampled. The collected muscle tissues were immediately placed in fixative solution for subsequent hematoxylin-eosin (HE) staining and muscle fiber analysis.

### 4.8. Western Blotting (WB)

Protein was extracted from CJ muscle tissue using RIPA dissolution buffer (G2002-100ML, Servicebio, Wuhan, Hubei, China), and the concentration was determined using the BCA protein detection kit (G2026-200T, Servicebio, China). Samples containing equal amounts of protein were separated via SDS-PAGE (G2181-2.5L, Servicebio, China) and then transferred to PVDF membranes (G6047-50-0.45, Servicebio, China). The membranes were incubated overnight with specific primary antibodies against the Ube3a protein (HUABIO, 1:1000, China) and lamin B1 protein (GB151802, Servicebio, 1:1000, China). The IgG secondary antibody (GB23303, 1:3000) was purchased from Servicebio (China) and incubated at room temperature for 1 h. Finally, an enhanced chemiluminescence (ECL) reagent (G2020-1, Servicebio, China) was used as the substrate for signal development. Detailed sampling and analysis time points are summarized in Table S10.

### 4.9. Immunofluorescence (IF)

CJ muscle tissue was fixed and embedded in paraffin, then sectioned. Paraffin sections were dewaxed and blocked with 3% hydrogen peroxide solution and 3% BSA (GC305006), respectively. The tissues were then washed with PBS (pH = 7.4) three times for 5 min each time. To identify the interference effect of *ube*3*a*-dsRNA, Ube3a primary antibody diluted at 1:1000 was added and incubated overnight at 4 °C. After the slides were washed with PBS three times, the HRP-conjugated secondary antibody matching the primary antibody species was added dropwise and incubated at room temperature for 50 min. After washing with PBS, the corresponding TSA (G1231, if488-Tyrosine, Servicebio, China) was added dropwise and incubated at room temperature in the dark for 10 min. After incubation, the slides were shaken and washed in TBST for 3 times, each time for 5 min. DAPI was added to the slides using DAPI staining solution (G1012, Servicebio, China), and then incubated at room temperature for 10 min before washing with PBS. After slightly drying the slices, the autofluorescence quencher (G1221, Servicebio, China) was added to the slices and incubated for 5 min, and then the slices were rinsed with running water for 10 min. Afterward, the slices were washed with PBS solution three times, each time for 5 min. Finally, the slices were blocked with anti-fade mounting medium (G1401, Servicebio, China). Results were observed and photographed by fluorescence microscopy. Three experimental fish from each experimental group were used to obtain 3 parallel slice results. Detailed sampling and analysis time points are summarized in Table S10. We used fish as the biological replicate and field of view and muscle fibers as the technical measurement units. Using Saiviewer 2.2.4 software, 20 visual fields of the staining image were captured in each slice result, and the positive area ratio (Ube3a-positive area/visual field was calculated based on the relative fluorescence intensity. All image scoring and quantification were conducted blindly, with sample group identities hidden to prevent subjective bias.

### 4.10. Muscle Fiber Density, Diameter, and Area Determination

The muscle tissues of six CJ fish were collected at the above 5 time points (0 d, 3 d, 7 d, 14 d, 21 d; Table S10), and then fixed with paraformaldehyde, diluted with ethanol, dehydrated, cleared with xylene, and embedded in paraffin. Subsequently, the tissues were transected to 5-μm thickness and mounted on slides. The slides were stained with hematoxylin and eosin, and then observed under a microscope (Eclipse Ci-L, Nikon Corporation, Tokyo, Japan). Muscle fiber density, diameter and area were determined following our previous research [5].

### 4.11. Data Analysis

The expression level of the target gene was normalized to the internal reference gene *β-actin*, and then statistically analyzed using SPSS Statistics v25.0 (SPSS Inc., Chicago, IL, USA) software. The Shapiro-Wilk test was used to verify the normality of data distribution prior to statistical comparison. The t-test was used to assess the significance of differences (*p* < 0.05) between samples. The results were plotted using Excel software, and all experimental data were expressed as mean ± standard error (Mean ± SEM).

## 5. Conclusions

In summary, the reduced locomotor capacity of CJ carp may be associated with the activation of the ubiquitin-proteasome pathway and decreased energy metabolism levels. We speculate that high expression of ubiquitination-related genes in muscles might promote protein degradation and suppress muscle fiber hypertrophy, while reduced expression of energy metabolism-related genes impairs energy production. Further in vivo interference experiments indicate that *ube*3*a* may be mainly regulated at the translational or protein stability level. The downstream target genes *stat*3 and *carm*1 exhibit distinct regulatory patterns, with *carm*1 exerting biological effects later than *stat*3. However, due to the absence of swimming performance assays in this study, the direct regulatory relationship between *ube*3*a* and fish locomotor capacity cannot be confirmed. Subsequent swimming performance tests are required to further elucidate the regulatory effect of *ube*3*a* on fish locomotion.

## 6. Limitations of the Study

First, the sample sizes for transcriptomic and proteomic sequencing were relatively small. Although the sampling age was the same, differences in body size among strains constituted a confounding factor. Furthermore, because locomotor behavior was not quantified, it was not possible to directly link gene expression differences to swimming performance. Second, different screening thresholds were used for the transcriptomic and proteomic analyses, and no unified multiple-testing correction was applied; therefore, these omics results are suitable only for screening candidate genes and proteins, rather than for drawing definitive conclusions. Third, the in vivo RNAi experiments used only the saline group as a control and lacked a control with random-sequence, non-targeting double-stranded RNA (dsRNA), making it impossible to fully distinguish the specific phenotype caused by *ube*3*a* knockdown from biological or immune responses induced by non-targeting dsRNA. Fourth, for the RNAi experiments, the effects of the aquarium, repeated sampling, and the treatment-by-time interaction were not fully accounted for; subsequent analyses will be conducted using two-way analysis of variance (ANOVA) or mixed-effects models with corrections for multiple comparisons.

## Figures and Tables

**Table 1:** Primer sequences.

Primer	Sequences (5′-3′)
*dsube*3*a*-1 F	TAATACGACTCACTATAGGGAGACTCAAGTGCGGAGAGTCTGG
*dsube*3*a*-1 R	GAGCCGGAAGAGGTAGCTTG
*dsube*3*a*-2 F	CTCAAGTGCGGAGAGTCTGG
*dsube*3*a*-2 R	TAATACGACTCACTATAGGGAGAGAGCCGGAAGAGGTAGCTTG

## Data Availability

The transcriptome sequencing data were submitted to the NCBI BioProject database under accession number PRJNA1275634 (https://www.ncbi.nlm.nih.gov/bioproject/PRJNA1275634/ (accessed on 12 July 2025). The proteomic sequencing data were submitted to the iProX database (https://www.iprox.org (accessed on 13 July 2025)) under accession number IPX0012287000.

## Supplementary Materials

The following supporting information can be downloaded at: https://www.mdpi.com/article/10.3390/ijms27219624/s1.
